# Cervical plexus anesthesia versus general anesthesia for anterior cervical discectomy and fusion surgery: A randomized clinical trial

**DOI:** 10.1097/MD.0000000000006119

**Published:** 2017-02-17

**Authors:** Hui Wang, Lei Ma, Dalong Yang, Tao Wang, Qian Wang, Lijun Zhang, Wenyuan Ding

**Affiliations:** a Department of Spine Surgery; b Financial Statistics Office, The Third Hospital of HeBei Medical University; c The Orthopaedic Department From First Hospital of Shijiazhuang, Shijiazhuang, China.

**Keywords:** cervical plexus anesthesia, general anesthesia, patient satisfaction

## Abstract

Both general anesthesia (GA) and cervical plexus anesthesia (CPA) can be used for anterior cervical discectomy and fusion (ACDF) surgery. The aim of this study was to evaluate the influence of anesthetic techniques on perioperative mortality and morbidity in patients undergoing cervical surgery.

From January 2008 to December 2015, 356 patients who underwent 1-level ACDF for cervical spinal myelopathy were prospectively reviewed. They were assigned to receive GA (group A) and CPA (group B). The analgesic efficacy of the block was assessed by anesthesia preparation time, the maximum heart rate, and mean arterial blood pressure changes compared with the baseline, time of postoperative revival, and duration of recovery stay. Duration of surgery, blood loss, and anesthesia medical cost were also recorded. Numerical rating scale (NRS) was used to evaluate pain at different time points. Postoperative nausea and vomiting (PONV) was assessed, and postoperative average administered dosages of meperidine and metoclopramide were also recorded. The spinal surgeon satisfaction, anesthetist satisfaction, and patient satisfaction were assessed.

Both the anesthesia induction time and postoperative revival time were longer in group A than that in group B; both the duration of surgery and recovery stay were also longer in group A than that in group B, whereas there was no difference in blood loss between the 2 groups. The average dosage of both meperidine and metoclopramide was more in group A than that in group B, and the anesthesia medical cost was greater in group A than that in group B. There were no significant differences in baseline data of systolic blood pressure, diastolic blood pressure, and heart rate between the 2 groups. But the intraoperative data of systolic blood pressure, diastolic blood pressure, and heart rate were higher/larger in group B than that in group A. In group A, there was no complaint of pain in the surgery procedure, but the pain increased after GA, with highest degree at 8 hours postoperation; then the pain degree decreased, and the NRS was 1 at 24 hours postoperation. In group B, intraoperative pain was NRS 4, and pain degree decreased from 4 hours postoperation; the NRS was 2 at 24 hours postoperation. The incidence of severe PONV was higher in group A than that in group B. There was no significant difference in the spinal surgeon satisfaction and anesthetist satisfaction for the anesthetic techniques. There was significant difference in patient satisfaction between the 2 groups, with high satisfaction for GA.

General anesthesia is superior to CPA in maintaining better intraoperative hemodynamic stability and providing high patient satisfaction with no intraoperative pain for patients receiving ACDF, but it entails longer surgery and anesthesia time, and requires more postoperative analgesic and anesthesia cost.

## 1 Introduction

Anterior cervical discectomy and fusion (ACDF) carries a significant risk of intraoperative spinal cord injury, with the potential of postoperative paralysis or even death. Various methods for intraoperative spinal cord function monitoring can be utilized, but the assessment of the patient's consciousness remains the easiest and most available method, requiring that the patient remains awake and is under regional anesthesia.^[1]^ However, most of the cervical decompression and fusion surgeries are performed under general anesthesia (GA), mainly because anesthetists are far more familiar with providing general over regional anesthesia. The regional anesthesia of cervical plexus nerves for cervical surgical procedures is a serious challenge for anesthesiologists, especially because of the complex innervation and proximity of many anatomically important and delicate structures.^[2]^

Several investigations have been performed to evaluate the usefulness and effectiveness of cervical plexus blocks (CPBs) in a variety of surgical procedures, such as thyroid and parathyroid surgery, carotid endarterectomy, and also vocal cord surgery.^[3–8]^ It is widely acknowledged that the regional anesthesia of CPBs has several unique advantages when compared with GA, such as better postoperative analgesia, faster recovery, lower costs, morbidity, and mortality rates.^[9]^ Hisham and Aina^[10]^ demonstrate that CPB is an effective alternative to GA for thyroid surgery and is reported to be associated with high levels of patient satisfaction and low morbidity. Calderon et al^[11]^ prove that CPB is an alternative to GA for carotid endarterectomy, and enjoys the advantages of the neurological clinical intraoperative evaluation, shorter hospitalization, fewer shunts, and lower incidence of cerebrovascular accident. To the best of our knowledge, no previous study has been reported focusing on the comparison of anesthetic effect and complications between GA and CPB in anterior cervical spine surgery. The purpose of this study is therefore to explore the influence of anesthetic techniques on perioperative mortality and morbidity in patients undergoing cervical surgery.

## 2 Materials and methods

### 2.1 Patients

This is a prospective study, and it was approved by the Institutional Review Board of the Third Hospital of HeBei Medical University before data collection and analysis. Inclusion criteria were as follows: patients underwent 1-level ACDF for cervical spinal myelopathy and gave their written informed consent for participation. Exclusion criteria were as follows: patients with known bleeding diathesis, history of allergy to local anesthetics, ossification of posterior longitudinal ligament, C1–2 and C2–3, local sepsis, or known diaphragmatic motion abnormalities.

From January 2008 to December 2015, 356 patients who met both the inclusion and exclusion criteria in our hospital were prospectively reviewed. Among them, 182 were female and 174 male, with mean age of 52.2 ± 9.8 years (range from 34 to 73 years). Forty-seven cases were of C3–4, 106 cases of C4–5, 139 cases of C5–6, and 64 cases of C6–7. Using a random number sequence and sealed envelopes, 356 patients were assigned to receive GA (group A, 169 patients) and cervical plexus anesthesia (CPA) (group B, 187 patients).

### 2.2 Anesthetic technique

All of the ACDF surgeries were carried out by the same surgical and anesthesia groups. The incision line was infiltrated with lignocaine 1% and adrenaline 1:100,000 in both the group A and group B.

In group A, patients were anesthetized with propofol, lidocaine, and fentany. Endotracheal intubation was facilitated with atracurium. Anesthesia was maintained with 1.2% isoflurane and nitrous oxide 50% in oxygen. Morphine was administered for intraoperative analgesia. The heart rate, systolic blood pressure, diastolic blood pressure, mean arterial blood pressure, and oxygen saturation were monitored throughout the operation using ECG, noninvasive blood pressure monitoring, and pulse oximetry. When the wound closure was done, the anesthetic drugs were discontinued after patients received 100% oxygen. Subsequently, neuromuscular blockade was reversed by using neostigmine and atropine. When patients had spontaneous respiration, pulse oximeter oxygen saturation more than 95%, end-tidal carbon dioxide 35 to 40 mm Hg, respiratory rate less than 30 per minutes, and tidal volume more than 5 mL/kg, the trachea was extubated and patients were transferred to the postanesthesia care unit (PACU), and when the patients had no pain, nausea, and vomiting, they were discharged from the PACU.

In group B, all the manipulation was performed on the right side. For deep block, a 23-gauge, short-beveled needle was inserted behind the lateral border of the sternocleidomastoid muscle, 3 cm distal to the mastoid process, then 10 mL of 0.5% ropivacaine was administered bilaterally (5 mL). The superficial block was performed by using the same needle inserted at the midpoint of the lateral border of the sternocleidomastoid muscle, then 10 mL of 0.5% ropivacaine was administered bilaterally (5 mL) to block the main branches of the plexus. The onset of action for this block is 10 to 15 minutes, and the first sign of nerve blockade is decreased sensation in the area of distribution of the respective components of the cervical plexus. All patients were positioned in neck extension for ease of surgery at the request of the surgeon. Oxygen was administered to all patients via a simple face mask. During surgery, oral communication with the patient was maintained at all times. Reports of pain at skin incision or closure by the patient prompted the surgeon to infiltrate lidocaine 2% within the surgical fields. The patients were transferred to the PACU after spontaneous respiration, pulse oximeter oxygen saturation more than 95%, end-tidal carbon dioxide 35 to 40 mm Hg, respiratory rate less than 30 per minute, and tidal volume more than 5 mL/kg. When patients had no pain, nausea, and vomiting, they were discharged from the PACU.

### 2.3 Evaluation of anesthetic effect and satisfaction

The analgesic efficacy of the block was assessed by anesthesia preparation time (the time from anesthetic preparation to anesthesia meeting the requirement of operation), the maximum heart rate, and mean arterial blood pressure (systolic blood pressure and diastolic blood pressure) changes compared with the baseline, time of postoperative revival (the time from end of operation to leaving the operation room), and duration of recovery stay (the time from arrival to the PACU to discharge from it). Duration of surgery was calculated as the time from beginning of surgery to the closure of wound by the last suture. Blood loss was monitored and recorded by calculating the volume of blood suctioned from the surgical field. Complications and anesthesia medical cost were also recorded.

For all the patients, the level of pain was evaluated by the numerical rating scale (NRS). Questions were asked immediately after surgery. Postoperatively, pain was also evaluated every 4 hours on the ward for 24 hours. If the patient's pain NRS was higher than 4 or they strongly required, rescue analgesics were administrated at 6-h intervals or longer. Postoperative total administered dosage of meperidine was recorded till 24 hours after surgery. Postoperative nausea and vomiting (PONV) for 24 hours was also assessed by the “PONV grade” as 1 = no nausea; 2 = mild nausea; 3 = severe nausea; 4 = retching and/or vomiting. Severe PONV was defined as grades 3 and 4.^[12]^ When severe PONV developed and lasted more than 10 minutes, rescue antiemetics were administrated with metoclopramide at 0.1 mg/kg.

For the spinal surgeons, postoperatively, they were asked to subjectively assess the operating field conditions using a 4-degree descriptive scale of very good, good, bad, and very bad. For the anesthetists, postoperatively, they were asked to subjectively assess the anesthesia procedure using a 4-degree descriptive scale of very good, good, bad, and very bad. The patients’ satisfaction were evaluated by the modified Patient Satisfaction Index (m-PSI) before discharge, with response of 1 or 2 considered to indicate a satisfied outcome, and a PSI response of 3 or 4 to indicate a dissatisfied outcome (Table 1).

**Table 1:**
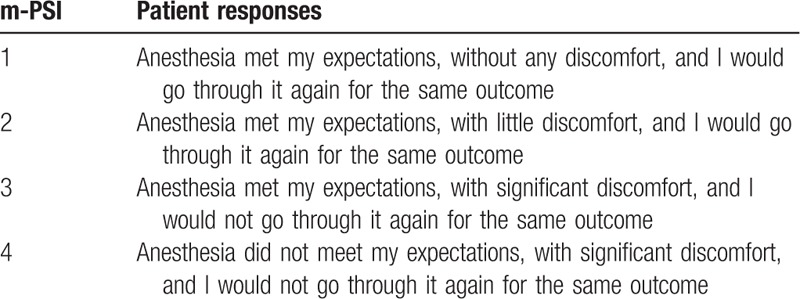
Modified Patient Satisfaction Index (m-PSI) for anesthesia techniques.

### 2.4 Statistics

Data were analyzed using Statistical Product and Service Solutions software (version 13; SPSS, Chicago, IL). The independent *t* test was used to evaluate numeric variables and chi-square test or nonparametric test was used to evaluate countable variables. Statistical significance was accepted at the 0.05 alpha level.

## 3 Results

At baseline, there were no significant differences between the 2 groups in age, sex, body mass index (BMI), cervical curvature index (CCI), and surgical level (Table 2).

**Table 2:**
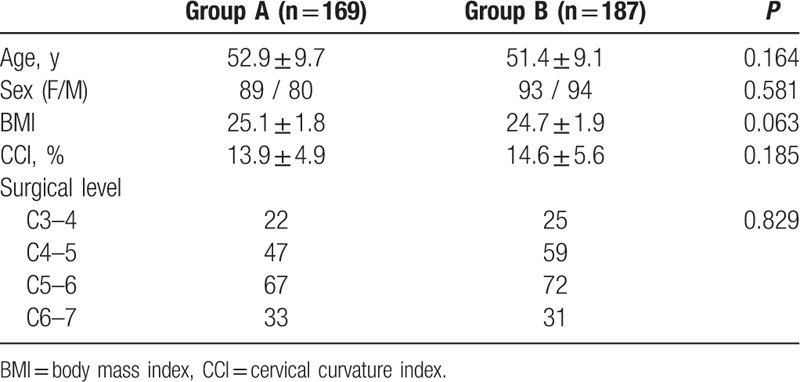
Patient demographic and baseline characteristics.

Both the anesthesia induction time and postoperative revival time were longer in group A than that in group B; both the duration of surgery and duration of recovery stay were also longer in group A than that in group B, whereas no difference in blood loss was found between the 2 groups. Both the average dosage of meperidine and average dosage of metoclopramide were more in group A than that in group B, and the anesthesia medical cost was greater in group A than that in group B. In group B, 3 patients experienced cervical nerve palsy, and recovered before discharge with neurotrophic drugs. Two patients experienced Horner syndrome (right side), and recovered at the first outpatient follow-up (3-month after operation) without intervention. One patient was converted to GA with intubation (Table 3).

**Table 3:**
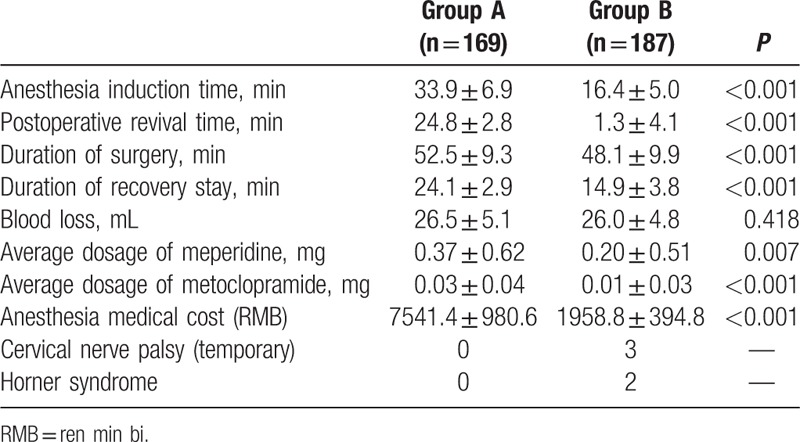
Patient anesthesia-related evaluation index.

There were no significant differences in baseline data of systolic blood pressure, diastolic blood pressure, and heart rate between the 2 groups. But the intraoperative data of systolic blood pressure, diastolic blood pressure, and heart rate were higher/larger in group B than that in group A (Table 4).

**Table 4:**
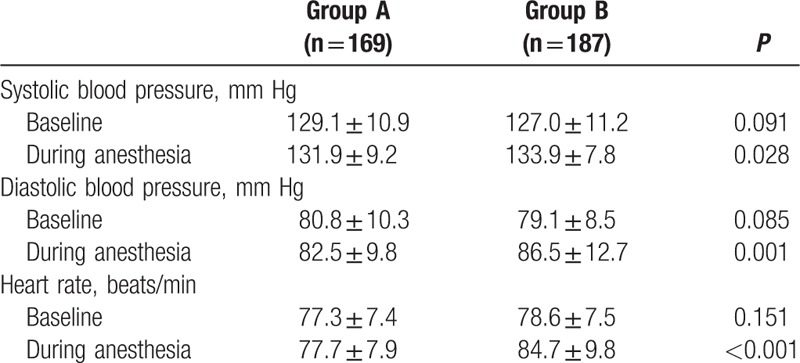
Perioperative hemodynamic stability.

**Figure 1:**
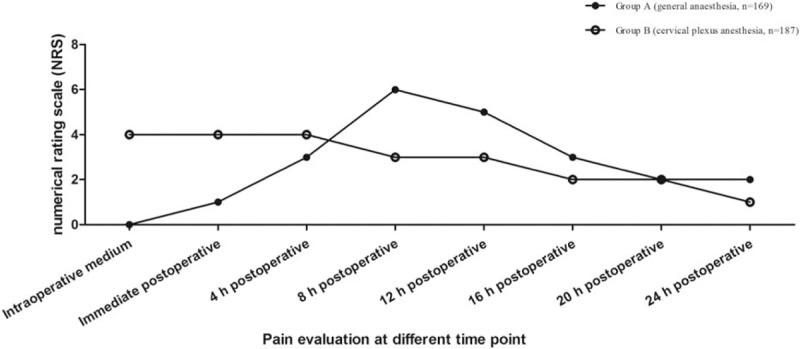
Pain evaluation by numerical rating scale (NRS) at different time points.

In group A, there was no complaint of pain in the surgery procedure, but the pain increased after GA, with highest degree at 8 hours postoperation; after that the pain degree decreased, and the NRS was 1 at 24 hours postoperation. In group B, intraoperative pain was NRS 4, and pain degree decreased from 4 hours postoperation; the NRS was 2 at 24 hours postoperation (Fig. 1).

**Figure 2:**
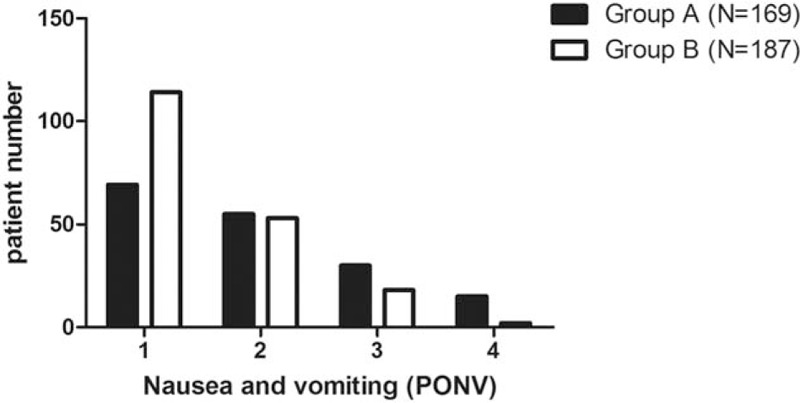
Nausea and vomiting (PONV) during the first 24 hours after operation. PONV = postoperative nausea and vomiting.

In group A, there were 69 patients with PONV 1, 55 patients with PONV 2, 30 patients with PONV 3, and 15 patients with PONV 4. In group B, there were 114 patients with PONV 1, 53 patients with PONV 2, 18 patients with PONV 3, and 2 patients with PONV 4. The incidence of severe PONV was higher in group A than that in group B (*χ*^2^ = 23.193, *P* < 0.001; Fig. 2).

**Figure 3:**
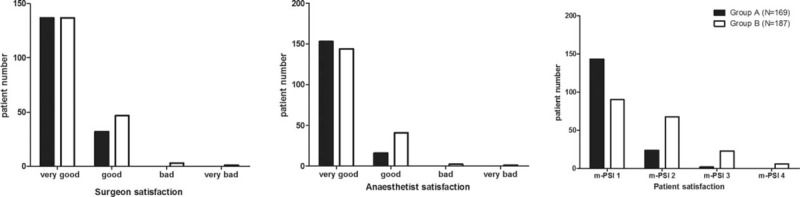
Surgeon, anesthetist, and patient satisfaction for anesthesia techniques.

In group A, for spinal surgeons, satisfaction was very good in 137 cases and good in 32 cases. For anesthetists, satisfaction was very good in 153 cases and good in 16 cases. In group B, for spinal surgeon, satisfaction was very good in 136 cases, good in 47 cases, bad in 3 cases, and very bad in 1 case. For anesthetists, satisfaction was very good in 143 cases, good in 41 cases, bad in 2 cases, and very bad in 1 case. There was no significant difference in surgeon satisfaction (Z = −1.604, *P* = 0.109) and anesthetist satisfaction (Z = −0.730, *P* = 0.465) between the 2 groups. In group A, satisfaction of m-PSI was 1 in 143 patients, 2 in 24 patients, and 3 in 2 patients. In group B, satisfaction of m-PSI was 1 in 90 patients, 2 in 68 patients, 3 in 23 patients, and 4 in 6 patients. There was significant difference in patient satisfaction between the 2 groups (*χ*^2^ = 22.914, *P* < 0.001) (Fig. 3).

## 4 Discussion

In the current study, we find that anesthesia induction time, postoperative revival time, and duration of recovery stay are longer in GA, and we assume that the difference is due to the inherent characteristics of anesthesia methods. For GA, induction procedure is defined as the period between the administration of induction agents and complete loss of consciousness. The commonly used induction agents include propofol, lidocaine, fentany, sodium thiopental, etomidate, and ketamine, which are more complex than CPA and needs more time to administer. Due to the effects of anesthetics, opioids, and muscle relaxants, the anesthetized patients will lose protective airway reflexes, airway patency, and sometimes a regular breathing pattern.^[13]^ To maintain a constant open airway, enable effective mechanical ventilation, and regulate breathing, an endotracheal tube is often adopted to ensure adequate gas exchange; this may increase the induction time compared with the CPA, which provides oxygen via a simple face mask. Moreover, difficult airway in GA is not common, but could inevitably increase the endotracheal intubation time.^[14,15]^ After termination of operation under GA, the anesthetic drugs were discontinued, and neuromuscular blockade was reversed by using neostigmine and atropine. Recovery of consciousness occurred when the concentration of anesthetics in the brain dropped below a certain level.^[13]^ For patients receiving CPA, the end of surgery means the end of anesthesia, without the procedure of consciousness recovery. More importantly, temporary neurologic phenomena always occur in the procedure of consciousness recovery, such as acute mental confusion, aphasia, or impairment in sensory or motor function. Cardiovascular events such as increased or decreased blood pressure, rapid heart rate, and other cardiac dysrhythmias are common during emergence from GA. ^[13]^ The potential risks associated with GA mentioned above may increase postoperative revival time and duration of recovery stay compared with CPA; most patients who receive CPA do not need to be transferred to the PACU, as they do not experience the induce unconsciousness and consciousness recovery procedure. The duration of surgery was longer in GA than that in CPA; this is more a consequence of surgeons’ consciousness that the patient is awake, resulting in faster work, than the consequence of anesthesia choice.^[16]^

General anesthesia maintains better intraoperative hemodynamic stability in systolic blood pressure, diastolic blood pressure, and heart rate when compared with CPA. To induce complete unconsciousness in GA, the anesthetics have varied sites of action and affect the central nervous system at multiple levels. Common areas within the central nervous system whose functions are interrupted or changed during GA include the cerebral cortex, thalamus, reticular activating system, and spinal cord.^[13]^ Satisfactory GA is manifested as the patient is unconscious and presents hemodynamic stability, no response to pain, skeletal muscles relax, stopping of vomiting and eye movements, and occurrence of respiratory depression. The cervical plexus is composed of the anterior rami of the 4 upper cervical spinal nerves, lies on the scalenus medius and levator anguli scapulae muscles, and deep to the sternocleidomastoid muscle, and gives off both superficial and deep branches. The superficial branches provide cutaneous innervation to the head and anterolateral neck, whereas the deep branches innervate the muscles of the anterior neck, the anterior and middle scalene, and the diaphragm.^[8,17]^ A combined method consisting of a superficial and a deep CPA could be used to achieve an effective cervical plexus nerve block for anterior cervical spine surgery; the patient maintains consciousness all through the surgery procedure, the pressure and dragging sensation of esophagus and trachea may produce anxiety and discomfort to make the patient uncooperative with the procedure, and may cause negative effects on intraoperative hemodynamic stability.

Under GA, the PONV can be caused by intubations, inhalation anesthetics, perioperative analgesics, and surgical manipulation.^[18]^ Sonner et al^[12]^ reported that 54% of the patients presented nausea and vomiting after thyroidectomy, with the incidence more common in women and in those who had inhalation anesthesia, and they scored PONV after thyroidectomy as grades 1 to 4 and defined severe PONV as grades 3 and 4.In the current study, incidence of severe PONV in GA was higher than that in CPA. Accordingly, the average dosage of metoclopramide in GA was higher than that in CPA. We suppose that the requirement of more anesthetic agents and endotracheal intubation in GA may account for the difference. Unconsciousness and no pain sensation in the surgical procedure is the main feature for GA, differing from CPA. In the current study, the NRS in GA was 0 all through the operation, but increased to the peak of 6 at 8 hours postoperation, and then decreased slowly. On the contrary, the NRS maintained the same of 4 all through the operation and continued to maintain the same 4 hours postoperation, and then decreased slowly. Accordingly, the average dosage of meperidine in GA was higher than that in CPA. Two possible explanations may account for the decreasing postoperative analgesic use in ACDF under CPA. First, the local anesthetics reduced the pain scores by directly preventing afferent nociceptive sensitization pathway. Second, the residue local anesthetics may have continued to play the role of pain relief at the first postoperative day.^[19,20]^

In the current study, there were no differences in spinal surgeon satisfaction and anesthetist satisfaction between GA and CPA, although the anesthesia medical cost was greater in GA, and the patient satisfaction for GA was higher than their satisfaction for CPA. Three possible reasons may account for the difference. First, CPA can be insufficient, because pain sensation caused by retractor tension or manipulation cannot always be treated sufficiently by additional wound-site infiltration. The quality of the plexus block has considerable influence on the patients’ satisfaction in those undergoing ACDF under CPA. Second, anesthesia-related complications, such as cervical nerve palsy and Horner syndrome, are not uncommon. Cause of Horner syndrome is the large accumulation of local anesthetic solution or the atypical proximal migration of the solution above the clavicle toward the supraclavicular paravertebral area. Although Horner syndrome had no clinical consequences, it may be described as an unpleasant side effect, and has the potential to lead to patient anxiety, discomfort, and dissatisfaction.^[21]^ Third, although the assessment of the patient's consciousness remains the easiest and most available method for intraoperative spinal cord function monitoring, staying awake during the operation is definitely an unpleasant experience for most of the patients undergoing ACDF under CPA.

There are some limitations to this study. First, this is a single-center study and only 356 patients were enrolled; selection bias may exist and multicenter large sample study is required. Second, only 1-level ACDF surgery was enrolled; whether the results apply to multilevel ACDF needs further study. However, we report the first prospective study to evaluate the influence of anesthetic techniques on perioperative mortality and morbidity in patients undergoing cervical surgery, and we find that GA is superior to CPA in maintaining better intraoperative hemodynamic stability, providing high patient satisfaction with no intraoperative pain for patients receiving ACDF, but it entails longer surgery and anesthesia time, and requires more postoperative analgesic and anesthesia cost. Our data provide reference in surgical planning and the selection of anesthesia methods for spinal surgeons, anesthetists, and patients receiving ACDF.
